# Supplementary material for: AI enabled sign language recognition and VR space bidirectional communication using triboelectric smart glove
Source: Nat Commun. 2021 Sep 10;12:5378. doi: 10.1038/s41467-021-25637-w (PMC8433305; doi:10.1038/s41467-021-25637-w)
Supplement: Supplementary file 1 — Supplementary Information [file 41467_2021_25637_MOESM1_ESM.pdf]

Supplementary Information for

AI enabled sign language recognition and VR space bidirectional communication using triboelectric smart glove

Feng Wen, Zixuan Zhang, Tianyiyi He, and Chengkuo Lee*

* Corresponding author: elelc@nus.edu.sg (C.L.)

Supplementary Note 1. The considerations behind the sensor distribution on gloves
Supplementary Note 2. The more detailed discussion about pros and cons of non-segmentation and segmentation methods
Supplementary Note 3. The accuracy performance of image recognition by comparing with sensor-based recognition system
Supplementary Figure 1. The detailed area information and channel label of sensors on gloves
Supplementary Figure 2. The photography of remaining 31 gestures and their corresponding triboelectric signals
Supplementary Figure 3. The train and validation accuracy increase with epochs
Supplementary Figure 4. The word frequency presented in the investigated 20 sentences
Supplementary Figure 5. The schematic diagram of segmentation
Supplementary Figure 6. The recognition result of three new sentences
Supplementary Figure 7. The radar comparison map of two methods
Supplementary Figure 8. The accuracy performance of image recognition when the brightness fades
Supplementary Table 1. CNN parameters
Supplementary Table 2. Detailed prediction of three new sentences
Supplementary Table 3. Benchmarking with other works

Supplementary Note 1. The considerations behind the sensor distribution on gloves

As depicted in Fig. 1b, the statistical analysis finds that the daily sign language involves three major motions, including elbow/shoulder motions, face muscle activities, and hand movements. The dominant hand motion accounts for 43%. Thus, the hand motion sensing is inevitable for sign language recognition. As shown in the enlarged pie chart in the right of Fig. 1b, the hand motions can be subdivided into four categories including finger bending (56%), wrist motion (18%), touch with fingertips (16%), and interaction with palm (10%). These detailed hand motions need sensors in different positions of hands to generate the essential correspondence.

Fig. 1c and Supplementary Fig. 1 show the triboelectric sensor is mounted on each finger for finger bending detection, while two sensors are put on wrists for wrist motion perception. In addition, the fingertips of index and middle of right hand are also in frequent use in daily used sign language and hence two sensors are located at fingertips. Meanwhile, signers often use their palms to interact with other parts of their body to convey richer information. But we nominally allocate only one sensor on the palm of left hand rather than two sensors one located on the left hand and one located on right hand. There are two major considerations behind such arrangement: (1) based on the minimalist design for reducing system complexity, we expect as few sensors as possible with the limit of capable of detect necessary hand motions. Thus, only one sensor is located on the left hand instead of one for left hand and one for right hand. (2) This sensor is attached on the left hand not right hand. Because the final status of most of gestures that involves palm end up on the palm of left hand, such as ‘Excuse’, ‘Medicine’, ‘Nice’, ‘School’, ‘Stop’ and ‘What’ shown in Supplementary Fig. 2. Hence one palm sensor on the left hand is reasonable to sense the interaction motions.

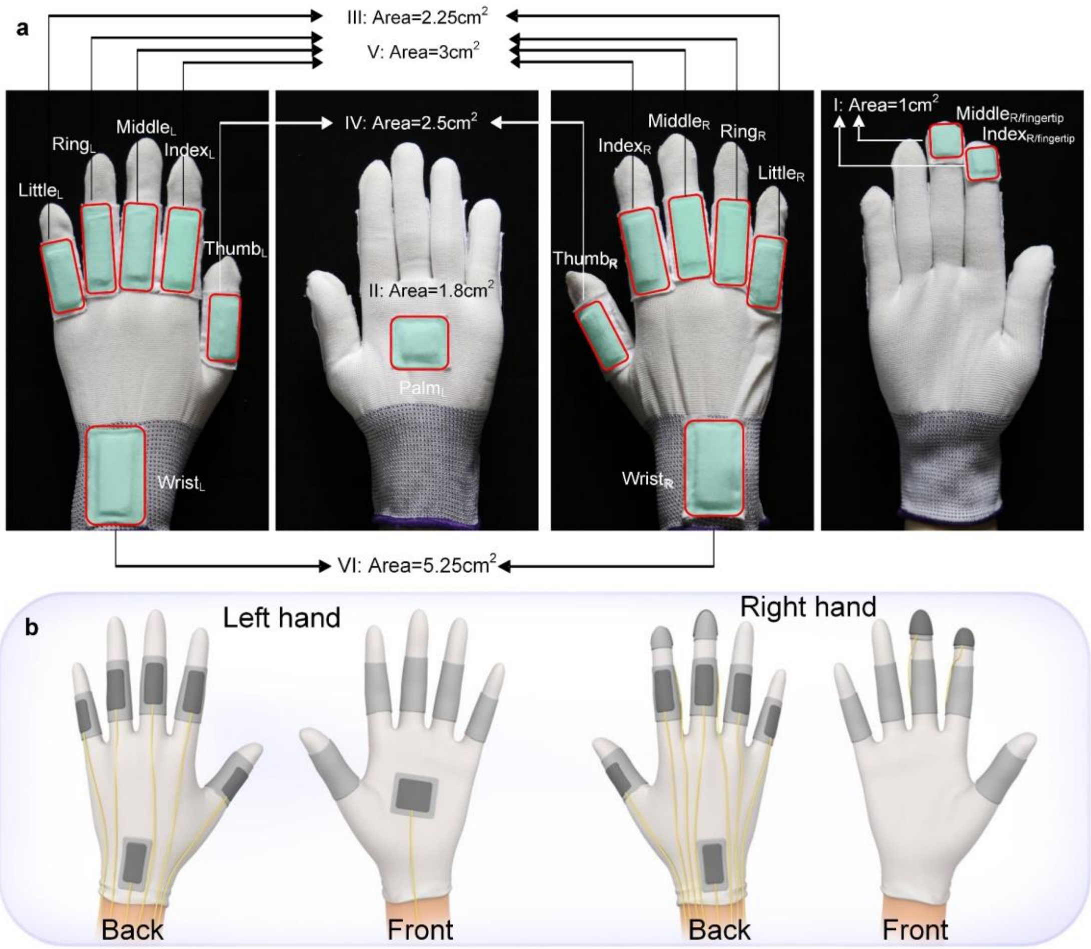

Supplementary Figure 1. The detailed area information and channel label of sensors on gloves. a Fabricated glove photos show detailed sensor area information and sensor channel label (corresponding with sensor output signal graphs) of sensors in a different position. **b** Schematic diagram of sensors on hand, corresponding with the photos of proposed gloves. The hand images are created by the authors via Blender. Photo credit: Feng Wen, National University of Singapore.

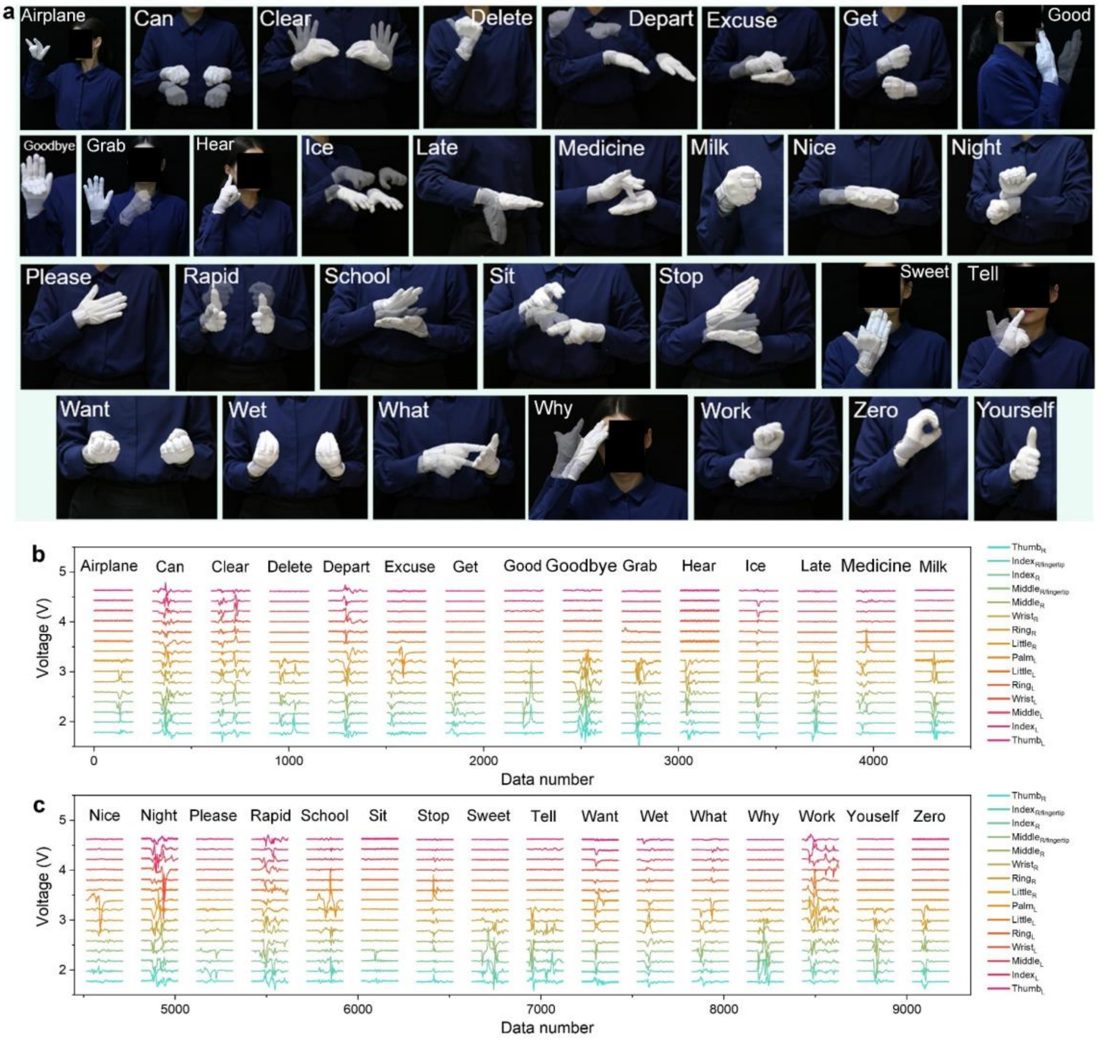

Supplementary Figure 2. The photography of remaining 31 gestures and their corresponding triboelectric signals. a Photography of the remaining 31 gestures. The opaque and translucent gesture images show the starting and final state of the gesture, respectively. These photos are of one of the authors. **b-c** Corresponding signals of these 31 gestures. Photo credit: Feng Wen, National University of Singapore.

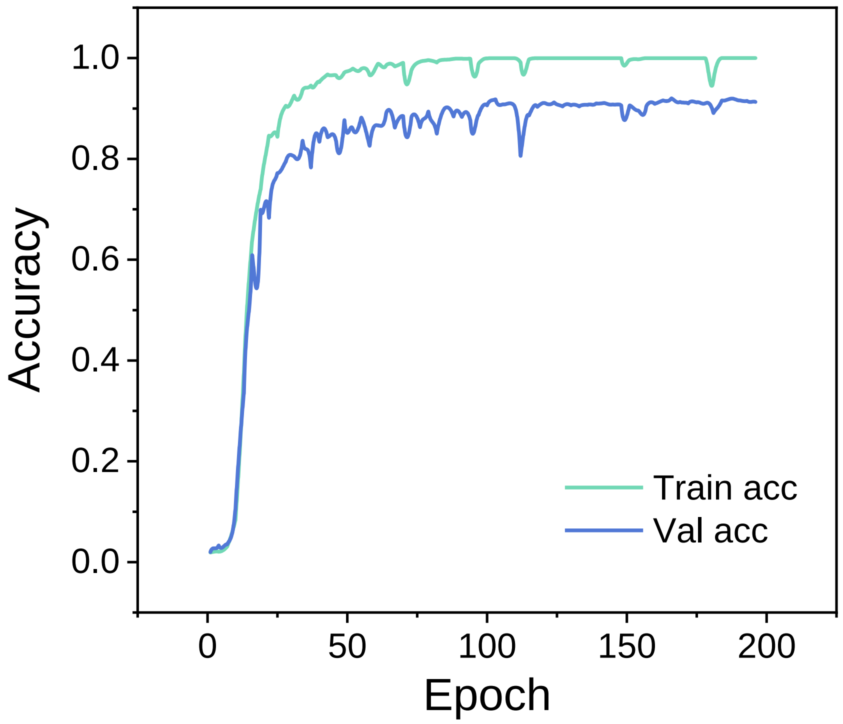

Supplementary Figure 3. The train and validation accuracy increase with epochs. After a small number of training epochs 50, the accuracy achieves an acceptable level, proving the good performance of the proposed CNN model for sign language recognition.

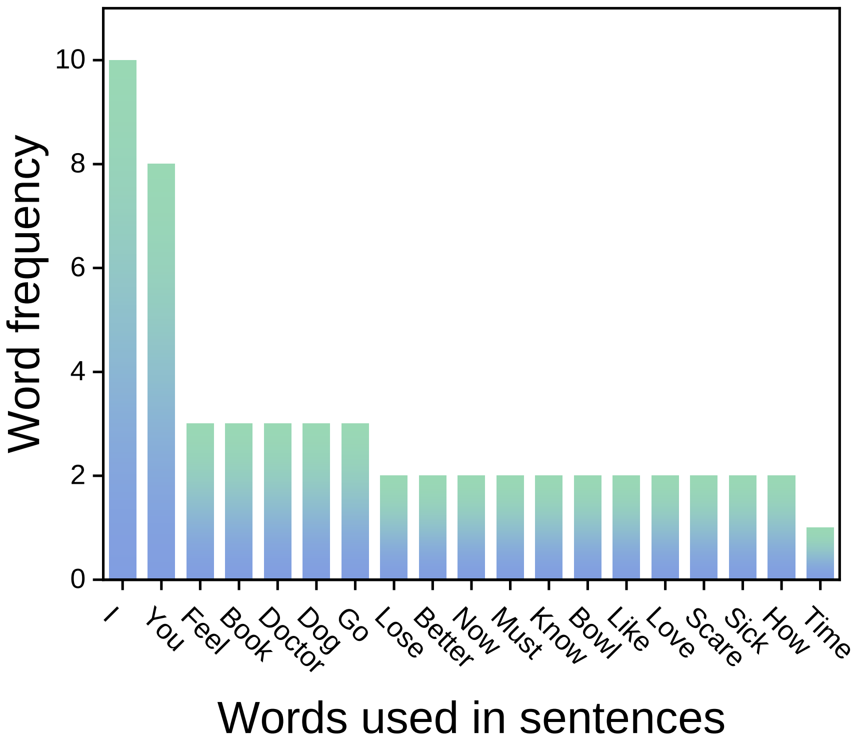

Supplementary Figure 4. The word frequency presented in the investigated 20 sentences. 19 Words are numbered from 0-18 according to the decreased usage frequency.

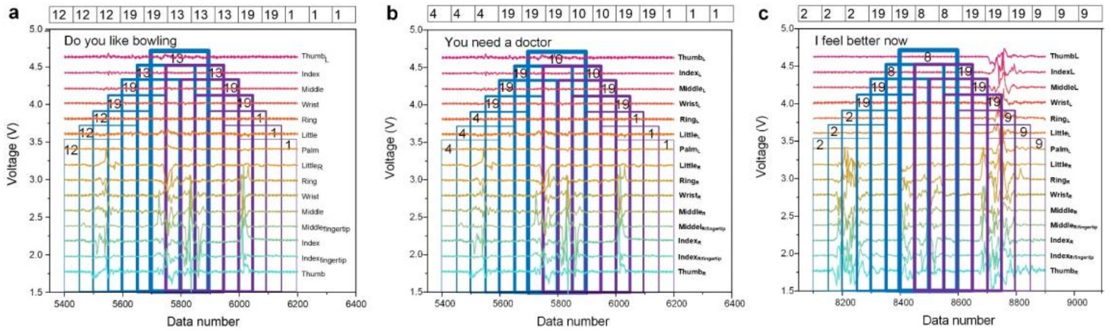

Supplementary Figure 5. The schematic diagram of segmentation. a ‘Do you like bowling’, **b** ‘You need a doctor’, and **c** ‘I feel better now’ as examples to show more detailed signal splitting process.

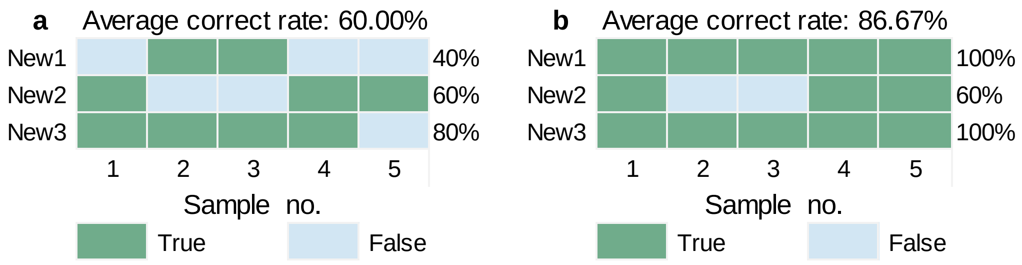

Supplementary Figure 6. The recognition result of three new sentences. a Using single classifier. **b** Using hierarchy classifier. The false prediction area is greatly reduced by using hierarchy classifier. Each sentence has been tested for five times with five samples.

Supplementary Note 2. The more detailed discussion about pros and cons of non-segmentation and segmentation methods

To illustrate the pros and cons of non-segmentation and segmentation methods, the detailed implementation of these two approaches should be discussed first. For non-segmentation method, each word or sentence is labeled as the independent individual. Then all the words and sentences will be separately trained in the neural network. Upon the completion of training, the CNN will recognize words and sentences independently. With such regime, either words or sentences essentially are different classes with respect to CNN's cognition, in which there is no built-up relationship between word units and sentences. For the strategy of segmentation, the data sliding window divides the entire sentence signal (800 data points) into fragments including intact word signals, incomplete word signals, and background signals. The label of fragment split from sentence signal is determined by the principal component. In other words, either word signal or background noise accounts for more than 50% of the sliding window size, and the label will be the number of corresponding words or 'empty' 19 as shown in Fig. 4a. Due to the specific size (200 data points) and sliding step (50 data points) of sliding window, the entire sentence signal is split into 13 fragments where each one is labeled with a number. Hence, the label of sentence will be a series of 13 numbers as illustrated in the top of Fig. 4b. Next, the sentence signals with the label of number sequences are included the dataset for training. The CNN classifier will go through all the fragments as well as the fragment sequence in sentences. Ultimately, both fragments and reversely reconstructed sentences by virtue of fragments can be correctly recognized. In particular, the CNN classifier is even endowed with the capability of recognizing never-seen sentences that comprise new-order word fragments, in which the never-seen sentences are not included in the dataset for training process and hence never learned by the neural network before.

Overall, as the radar map of comparison in Supplementary Fig. 7 shows, the non-segmentation approach possesses better performance in the aspect of recognition accuracy either for words or sentences. However, two following limitations of this means may compromise the universality and practicality of the whole system. Above all, owing to the independence of words and sentences, the CNN classifier cannot identify the new sentence although words in the sentence are seen before and only combined in a new order. In addition, when expanding sentence database, the labor-intensive data collection of new sentences and successive training are unavoidable. This kind of independence also leads to increased effort on data collection of words since the CNN model cannot extract and recognize the word signals in the sentence. Regarding the segmentation method, in addition to identifying existing sentences in the dataset, the CNN classifier enables the recognition of new sentences. These new sentences comprise new-order word series that are different from the order of existed sentences in the dataset. Nevertheless, the segmentation introduces a large amount of random and irregular 'empty' signals. It sacrifices the recognition accuracy for both words and sentences. Further research efforts could be committed to optimizing the algorithm framework and improve the recognition accuracy.

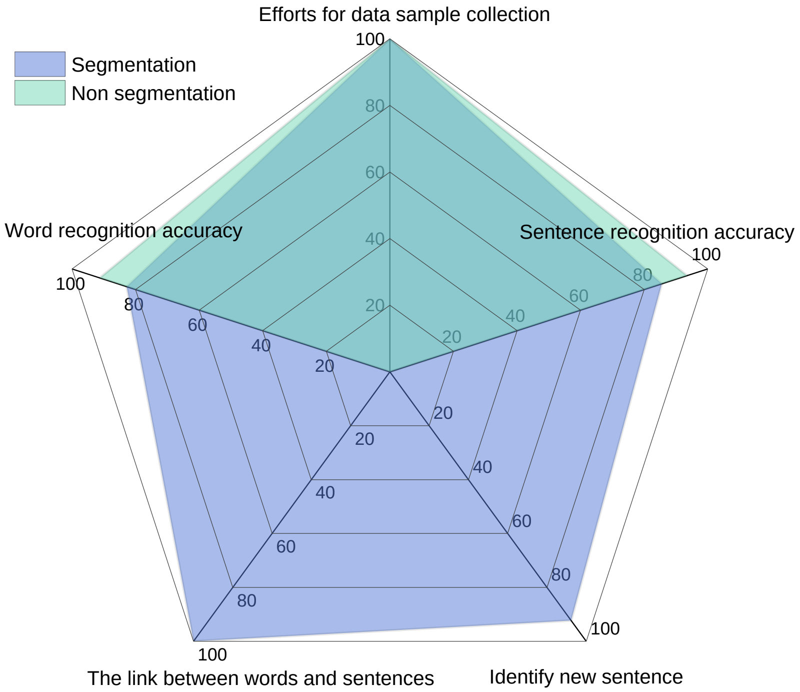

Supplementary Figure 7. The radar comparison map of two methods. Comparing non-segmentation and segmentation recognition methods based on their pros and cons.

Supplementary Note 3. The accuracy performance of image recognition by comparing with sensor-based recognition system

To clarify the advantages of sensor-based system, the additional test about the accuracy performance of visual images for gesture recognition is carried out. The recognition results of six representative gestures are shown in Supplementary Fig. 8 with varying light conditions (493, 275 and 13 lux). For each light condition, 50 trials of each gesture (300 trials in total) are carried out for image-based recognition. Supplementary Fig. 8b(i-iii) indicate a dramatically decayed recognition accuracy from 98.33% to 58.33% when the room light fades. The efficiency of visual images/videos recognition is well-known limited by the environmental interferences such as occlusions and especially light conditions. In addition, sign language involves the upper limbs as well as human faces. When the image-based system captures gesture information, the exposure of facial information to camera may arise the issue of privacy disclosure.

For sensor-based human gesture recognition system, wearable sensors, are typically less bulky, flexible and provide an intimate contact with the user for high-quality data acquisition and high-accurate recognition that is comparable with its image system counterpart. The sensor-based system is considered as one of approaches to overcome the drawbacks of image recognition. On the one hand, such sensor-based systems are not affected by varying luminance and can work well even under entirely dark condition with higher environmental tolerance. On the other hand, they can mitigate the privacy issue in cost-effective way owing to no need for individual information collection such as facial characteristics.

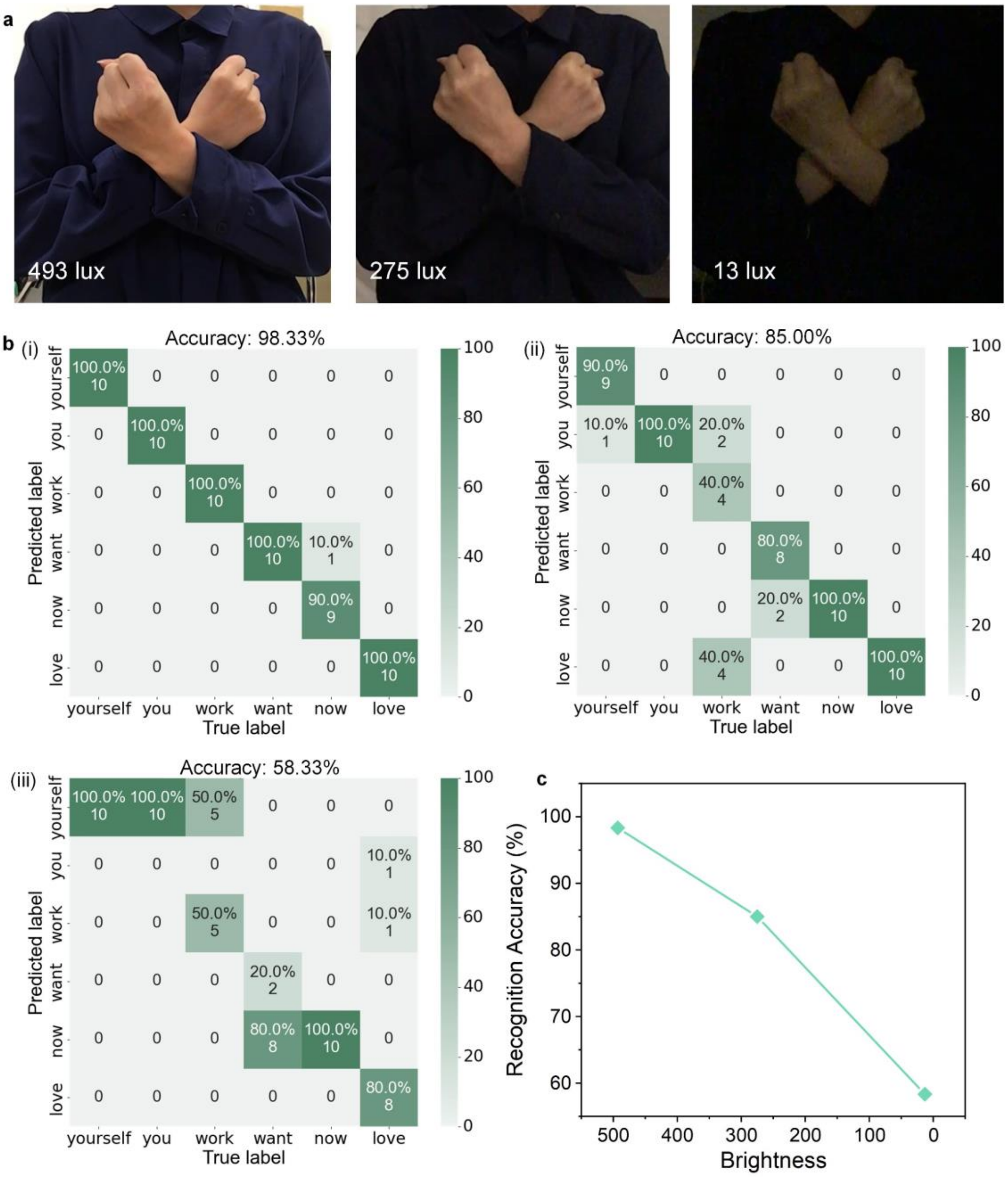

Supplementary Figure 8. The accuracy performance of image recognition when the brightness fades. a Gesture image of ‘Love’ under three light conditions. **b** (i-iii) Accuracy of the image recognition under different light conditions (493, 275 and 13 lux). These photos are of one of the authors. **c** Accuracy degradation with decreased brightness. Photo credit: Feng Wen, National University of Singapore.

Supplementary Table 1. **CNN parameters.** The detailed parameters for constructing Convolutional Neural Network (CNN).

No	Layer Type	No. of Filters	Kernel/ Pool Size	Stride	Input Size	Output Size	Padding
1	Convolution 2	64	5	1	(None, 100, 32)	(None, 100, 64)	same
2	Max Pooling 2		2	2	(None, 100, 64)	(None, 50, 64)	same
3	Convolution 3	128	5	1	(None, 50, 64)	(None, 50, 128)	same
4	Max Pooling 3		2	2	(None, 50, 128)	(None, 25, 128)	same
5	Convolution 4	256	5	1	(None, 25, 128)	(None, 25, 256)	same
6	Max Pooling 4		2	2	(None, 25, 256)	(None, 13, 256)	same
7	Convolution 5	512	5	1	(None, 13, 256)	(None, 13, 512)	same
8	Max Pooling 5		2	2	(None, 13, 512)	(None, 7, 512)	same
9	Flatten				(None, 7, 512)	(None, 3584)	same
10	Dense (500)				(None, 3584)	(None, 500)	
11	Dense (50)				(None, 500)	(None, 50)	

Supplementary Table 2. Detailed prediction of three new sentences. The predicted and true labels of single classifier and hierarchy classifier for three new/never-seen sentence recognition with numbers in red standing for wrong prediction.

Sentence	Sample	True label	Single classifier predicted label	Hierarchy classifier predicted label
New1	1	[0 0 19 19 19 7 7 19 19 19 5 5 5]	[0 0 19 19 8 7 7 19 19 19 5 5 5]	[0 0 19 19 19 7 7 19 19 19 19 5 5]
	2	[0 0 19 19 7 7 7 19 19 19 5 5 5]	[0 0 19 11 7 7 7 19 19 19 5 5 5]	[0 0 19 19 7 7 7 19 19 19 5 5 5]
	3	[0 0 19 19 19 7 7 19 19 19 19 5 5]	[0 0 19 19 6 7 7 19 19 19 19 5 5]	[0 0 19 19 19 7 7 19 19 19 19 5 5]
	4	[0 0 19 19 07 7 7 19 19 19 5 5 5]	[0 0 19 19 7 7 19 19 19 19 5 5 5]	[0 0 19 19 7 7 19 19 19 19 5 5 5]
	5	[0 0 19 19 19 7 7 19 19 19 19 5 5]	[0 0 19 19 19 7 7 7 19 19 19 5 5]	[0 0 19 19 19 7 7 19 19 19 19 5 5]
New2	1	[0 0 0 19 19 19 4 4 4 19 19 19 19]	[19 0 0 0 19 19 4 4 4 19 19 19 19]	[0 0 0 0 19 19 4 4 4 19 19 19 19]
	2	[0 0 0 19 19 19 19 4 4 19 19 19 19]	[0 0 0 19 19 19 4 4 4 19 19 19 19]	[0 0 0 19 19 19 4 4 4 19 19 19 19]
	3	[0 0 0 19 19 19 19 4 4 19 19 19 19]	[0 0 0 19 19 19 4 4 4 4 19 19 19]	[0 0 0 19 19 19 19 4 4 4 19 19 19]
	4	[19 0 0 0 19 19 19 4 4 4 19 19 19]	[19 0 0 18 19 19 19 4 4 4 19 19 19]	[1 0 0 0 19 19 19 4 4 4 19 19 19]
	5	[19 0 0 0 19 19 19 19 19 4 4 4 19]	[19 3 13 13 19 19 19 19 19 4 4 4 19]	[19 0 18 19 19 19 19 19 19 4 4 4 19]
New3	1	[17 17 19 19 19 1 1 1 19 19 2 2 2]	[17 17 17 19 19 1 1 0 19 19 2 2 2]	[17 17 19 19 19 1 1 1 19 19 2 2 2]
	2	[17 17 17 19 19 1 1 1 19 19 2 2 2]	[17 17 17 7 1 1 1 1 19 19 2 2 2]	[17 17 17 19 19 1 1 1 19 19 2 2 2]
	3	[17 17 17 19 1 1 1 19 2 2 2 19 19]	[17 19 17 19 1 1 1 19 2 2 2 19 19]	[17 17 17 19 1 1 1 19 2 2 2 19 19]
	4	[17 17 17 19 19 1 1 1 19 2 2 2 19]	[17 17 17 19 1 1 1 1 19 2 2 2 19]	[17 17 17 19 1 1 1 1 19 2 2 2 2]
	5	[17 17 17 19 19 1 1 1 19 2 2 2 19]	[17 19 17 7 19 1 1 1 19 2 2 2 19]	[17 19 19 19 1 1 1 1 19 2 2 19 19]

Supplementary Table 3. Benchmarking with other works. The benchmarking table for comparing with other similar works.

Mechanism	Device	Method	Sensor/hand	Gesture	Sentence	Recognize new sentence	Self-powered	Mutual interaction	Ref.
Piezoresistive	Armband	SVM	3	8 letters	-	-	×	×	1
Piezoresistive	Radar+wristband	HSVM	5	4 letters	0	×	×	×	2
Resistive	Finger sensing	SVM	5	10 letters	0	×	×	×	3
Resistive	Glove	BNN	9	10 letters	0	×	×	×	4
Resistive	Elastomer Glove	Amplitude	5	5	-	-	×	×	5
Resistive	Glove	Amplitude	5	9 words	0	×	×	×	6
Resistive	Glove	Amplitude	9	26 letters	0	×	×	×	7
Capacitive	Glove	Amplitude	5	5 numbers	-	-	×	×	8
Capacitive	Glove	Amplitude	5	36 letters	0	×	×	×	9
Capacitive	Wristband	Amplitude	15	15 words	0	×	×	×	10
Capacitive	Glove	XRF	16	10 numbers	0	×	×	×	11
Capacitive	Finger sensing	Amplitude	4	6 numbers	-	-	×	×	12
Ionic	Glove	Amplitude	8	6 words	0	×	√	×	13
Piezoelectric	Glove	Amplitude	5	6 numbers	0	×	√	×	14
Triboelectric	Glove	Amplitude	5	6 numbers	-	-	√	×	15
Triboelectric	Glove	Amplitude	6	7 numbers	-	-	√	×	16
Triboelectric	Glove	Amplitude	5	5 letters	0	×	√	×	17
Triboelectric	Glove	SVM	5	11 letters	0	×	√	×	18
Triboelectric	Glove	CNN	7	50 words	20	√	√	√	*

Note: (H)SVM: (hierarchical) support vector machine; BNN: binary neural network; XRF: extremely randomized trees; CNN: convolutional neural network. (*This work)

References

1. Esposito, D. *et al*. A piezoresistive array armband with reduced number of sensors for hand gesture recognition. *Front. in neurorob.* **13**, 114 (2020).
2. Liang, X., Li, H., Wang, W., Liu, Y., Ghannam, R., Fioranelli, F., & Heidari, H. (2019). Fusion of wearable and contactless sensors for intelligent gesture recognition. *Adv. Intell. Syst.* **1**, 1900088.
3. Li, L., Jiang, S., Shull, P. B., & Gu, G. SkinGest: artificial skin for gesture recognition via filmy stretchable strain sensors. *Adv. Robot.* **32**, 1112-1121 (2018).
4. Fan, T., *et al*. Analog Sensing and Computing Systems with Low Power Consumption for Gesture Recognition. *Adv. Intell. Syst.* **3**, 2000184 (2021).
5. Muth, J. T. *et al*. Embedded 3D printing of strain sensors within highly stretchable elastomers. *Adv. Mater.* **26**, 6307-6312 (2014).
6. Ambar, R. *et al*. Development of a wearable device for sign language recognition. *J. Phys. Conf. Ser.* **1019**, 012017 (2018).
7. O'Connor, T. F. *et al*. The language of glove: wireless gesture decoder with low-power and stretchable hybrid electronics. *PLOS One* **12**, e0179766 (2017).
8. Shintake, J., Piskarev, E., Jeong, S. H., & Floreano, D. Ultrastretchable strain sensors using carbon black-filled elastomer composites and comparison of capacitive versus resistive sensors. *Adv. Mat. Techno.* **3**, 1700284 (2018).
9. Abhishek, K. S., Qubeley, L. C. F. & Ho, D. Glove-based hand gesture recognition sign language translator using capacitive touch sensor. *2016 IEEE Int. Conf. Electron Devices and Solid-State Circuits,* pp. 334-337 (2016).
10. Truong, H. *et al*. Cap-band: Battery-free successive capacitance sensing wristband for hand gesture recognition. *In Proc. of the 16th ACM Conf. on Embedded Networked Sens. Syst.*, pp. 54-67 (2018).
11. Pan, J. *et al*. A wireless multi-channel capacitive sensor system for efficient glove-based gesture recognition with AI at the edge. *IEEE Trans. on Circuits and Syst. II: Express Briefs* **67**, 1624-1628 (2020).
12. Bartlett, M. D., Markvicka, E. J., & Majidi, C. Rapid fabrication of soft, multilayered electronics for wearable biomonitoring. *Adv. Funct. Mater.* **26**, 8496-8504 (2016).
13. Zhao, J. *et al*. Passive and space-discriminative ionic sensors based on durable nanocomposite electrodes toward sign language recognition. *ACS Nano* **11**, 8590-8599 (2017).
14. Fuh, Y. K., & Ho, H. C. Highly flexible self-powered sensors based on printed circuit board technology for human motion detection and gesture recognition. *Nanotechnology* **27**, 095401 (2016).
15. Lai, Y. C. *et al*. Single-thread-based wearable and highly stretchable triboelectric nanogenerators and their applications in cloth-based self-powered human-interactive and biomedical sensing. *Adv. Funct. Mater.* **27**, 1604462 (2017).
16. He, Q. *et al*. An all-textile triboelectric sensor for wearable teleoperated human–machine interaction. *Journal of Materials Chemistry A* **7**, 26804-26811(2019).
17. Maharjan, P. *et al*. A human skin-inspired self-powered flex sensor with thermally

embossed microstructured triboelectric layers for sign language interpretation. *Nano Energy* **76**, 105071 (2020).

18. Zhou, Z. *et al*. Sign-to-speech translation using machine-learning-assisted stretchable sensor arrays. *Nat. Electron.* **3**, 571-578 (2020).
