# Supplementary material for: AI enabled sign language recognition and VR space bidirectional communication using triboelectric smart glove
Source: Nat Commun. 2021 Sep 10;12:5378. doi: 10.1038/s41467-021-25637-w (PMC8433305; doi:10.1038/s41467-021-25637-w)
Supplement: Supplementary file 3 — Description of Additional Supplementary Files [file 41467_2021_25637_MOESM3_ESM.pdf]

Description of Additional Supplementary Files

File Name: Supplementary Movie 1
Description: Recognition of words in sign language

File Name: Supplementary Movie 2.
Description: Recognition of sentences in sign language

File Name: Supplementary Movie 3.
Description: The sign language recognition and communication system for interaction of speech-impaired user and non-signer
